# Nurses' Knowledge, Practice, and Associated Factors with Enteral Nutrition in Adult Intensive Care Units of Public Hospitals

**DOI:** 10.4314/ejhs.v32i2.23

**Published:** 2022-03

**Authors:** Tsige Hadera, Tigist Worku, Wagari Tuli

**Affiliations:** 1 Department of Nursing, College of Health Sciences, Mekelle University, Mekelle, Ethiopia; 2 Department of Emergency Medicine, College of Health Sciences, Addis Ababa University, Addis Ababa, Ethiopia; 3 Department of Emergency Medicine, College of Health Sciences, Addis Ababa University, Addis Ababa, Ethiopia

**Keywords:** Adult ICU, Enteral Nutrition, ICU nurse, Knowledge, practice

## Abstract

**Background:**

In critically ill patients, enteral nutrition is recommended as a route for nutrient delivery. Nurses' knowledge and practice of enteral nutrition influence patients' clinical outcomes. Therefore, this study sought to assess nurses' knowledge, practice, and associated factors regarding enteral nutrition in adult intensive care unit patients in public hospitals in Addis Ababa, Ethiopia.

**Methods:**

A cross-sectional study design was used to collect data from 196 nurses working in public hospitals in Addis Ababa from April 11 to April 30, 2020. The data were entered into Epi Data version 3.1 and analyzed with SPSS version 21. The correlation between independent variables and dependent variables was estimated using bivariate and multivariate logistic regression at a 95% confidence level.

**Results:**

The level of inadequate knowledge and poor practice of nurses relating to enteral nutrition was 67.7% and 53.8%, respectively. Bachelor's degree holders were less likely to be knowledgeable (AOR= 0.24, 95% CI: (0.61, 0.93)). Nurses' practice about enteral nutrition was significantly associated with nurses' age (AOR = 0.023, 95 % CI: (0.001,0.52), nurses receiving training on enteral nutrition (AOR = 1.951, 95 % CI: (0.06, 0.60)), and nurses from ICUs having a guideline and protocol on enteral feeding practice (AOR = 3.401, 95 % CI: (1.186, 9.789).

**Conclusions:**

In the study, it was revealed that a substantial proportion of nurses had inadequate knowledge of enteral nutrition and practiced poor enteral nutrition.

## Introduction

In the intensive care unit (ICU), nutrition is one of the most important aspects of medical care for critically ill patients (1–4). A high prevalence of morbidity and mortality is associated with malnutrition, affecting up to 40% of hospitalized patients (5–7). Enteral nutrition (EN) is a delivery system that supplies all the essential nutrients - including water and minerals - into the gastrointestinal tract (by mouth or tube) and then into the stomach, duodenum, or jejunum (8,9).

The Canadian Critical Care Practice Guidelines (CCPGs, 2013) and the American Society of Parenteral and Enteral Nutrition (ASPEN, 2009) recommended that EN is the preferred feeding method for critically ill patients due to its costeffectiveness, prevention of intestinal mucosal atrophy, and maintenance of intestinal immunity through gut-associated lymphoid tissue (9, 10).

In patients with functional gastrointestinal tracts, EN is indicated when oral nutritional intake is inadequate to meet estimated nutritional needs (3, 6). The use of EN should begin within the first 24 to 48 hours of admission for patients who receive ventilator support and have stable hemodynamic states with an adequate total caloric intake of 20 to 25 calories per kilogram of body weight for most adults in the ICU (8, 11, 12). It is crucial to consider the underlying medical condition, nutritional status, and available routes of nutrient delivery when determining the type and amount of nutritional support (13).

The ICU nurses play an important role in maintaining the daily nutritional status of patients. To prevent complications related to enteral feeding and improve outcomes, effective nursing practices such as the use of prokinetic agents, decreasing feeding rate, measurement of gastric residual volume, and maintaining the correct positioning of patients are required (14).

Although few data are available on nursing knowledge and practice regarding nutritional feeding in ICU, some studies have shown that nurses working in ICU have inadequate knowledge and poor practice that leads to the highest prevalence of malnutrition among hospitalized patients (15–18).

In some studies, factors such as lack of guidelines and protocols on EN, no established nutrition committee, a shortage of feeding tubes, patient characteristics such as refusal of tube feeding, differences in nurses' characteristics, and the environment where tube feeding practice is conducted may affect nurses' knowledge and their practice (19).

The investigators are unaware of any study conducted on nurses' knowledge, practice, and factors associated with enteral nutrition in ICUs of public hospitals in Addis Ababa, Ethiopia. So, this study aimed at assessing nurses' knowledge, nurses' practice, and associated factors with enteral nutrition in ICU of public hospitals in Addis Ababa, Ethiopia.

## Methods and Materials

**Study area and period**: The study was conducted from April 11 to April 30, 2020, in ten public hospitals in Addis Ababa, the capital city of Ethiopia. Those public hospitals are Tikur Anbessa Specialized Hospital (TASH), St. Paul Millennium Medical College and Hospital (SPMMCH), Addis Ababa Burn, Emergency and Treatment (AaBET) Hospital, Yekatit 12 Hospital, Tirunesh Beijing Hospital (TBH), Ras Desta Damtew Hospital (RDDH), Zewditu Memorial Hospital (ZMH), Menelik II Hospital, All Africa Leprosy, Tuberculosis, Rehabilitation, and Training Center (ALERT) Hospital and St Peter Hospital. The total number of ICU nurses in those public hospitals is 332.

**Study design**: A cross-sectional quantitative study design was conducted to assess knowledge, practice, and factors associated with enteral nutrition among nurses working at ICUs of public hospitals in Addis Ababa, Ethiopia.

**Source population**: All nurses who were working at ICUs of public hospitals in Addis Ababa were taken as the source population.

**Study population**: Nurses who were on the duty during data collection time and who fulfilled the inclusion criteria were our study population.

**Inclusion criteria and exclusion criteria**: Those nurses who were present during the study period and volunteer to participate in the study were included whereas those did not available (annual leave, maternal leave) during the study period were excluded from the study.

**Figure 1:**
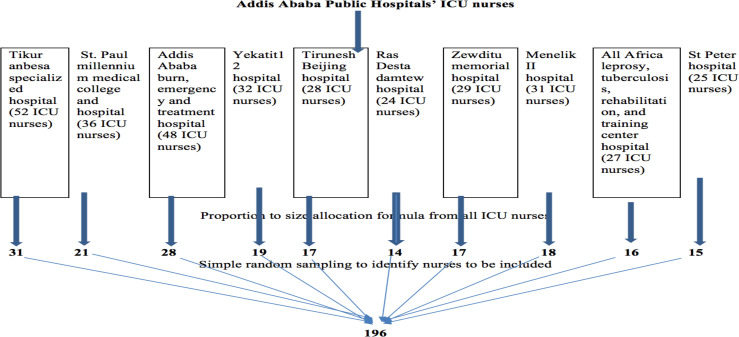
Schematic presentation of sampling procedure for the study participants, 2020.

**Sample size and sampling procedure**: Sample size (n) was determined based on a single proportion formula with the following assumption. Since there was no similar study done in Ethiopia, we took the prevalence of nurses' knowledge and nurses' practice on enteral nutrition as 50%. The level of confidence (α) was taken at 0.05 (Z (1-α/2) =1.96); the margin of error was taken as 0.05. By consideration of the 10% non-response rate, the final sample size (nf) of this study was 196. Also, the number of study units to be sampled from each ICU was determined using the proportion to size allocation formula. Lists of nurses were taken from each unit of hospitals and simple random sampling was used to select respondents from each ICU of the public hospitals in Addis Ababa (Figure 1).

**Data collection techniques and instruments**: A pre-tested, structured, self-administered questionnaire was used to collect data from study subjects. The questionnaire was developed after reviewing different kinds of literature (18, 20, 21) and some modification was done by different experts on the area from the nursing and nutrition departments of Addis Ababa University. The questionnaire was divided into four sections: demographic of respondents, nurses' knowledge, nurses' practice, and challenges toward delivering enteral nutrition. Demographic data included sex, age, level of education, and work experience of nurses. Nurses' knowledge about EN consisted of sixteen questions with three options (Yes, No and I don't know). One point was given for each correct answer; and for all other responses, zero points were assigned. The total score for knowledge ranged from zero to sixteen with high scores indicating adequate knowledge of EN. Nurses' practice about EN consisted of six questions with 4-point Likert scale questions ordered as never, sometimes, almost always, and always. One point was allocated to always and zero points for all other answers. The total score for practice ranged from zero to six with high scores indicating good practice. Challenges of nurses to apply practice on EN consisted of five questions labeled as dichotomy items (Yes/No). A score of one was given to answers that reflected challenges of delivering EN, and a score of zero was given to answers that reflected no challenges of delivering EN. Data collectors (one nurse from each unit of ICU) were experienced nurses who were selected according to their previous experiences of data collection.

**Data entry, process and analysis**: The data were checked for completeness and consistency by the principal investigator; then cleaned and coded for entry. The coded data were entered into epi data version 3.1 and exported to SPSS version 21.0 for analysis. Frequency and percentage were used to summarize the findings while tables and graphs were used to present the data. Bivariate logistic regression was used to show the association of each independent variable with dependent variables. First, the crude odds ratio (COR) of all independent variables on knowledge and practice of enteral feeding was calculated at a 95% confidence interval (CI), and all variables with a p-value of <0.25 were considered for multivariable logistic regression to control the effect of other confounders. Then, the significance level was set at P< 0.05.

**Ethics approval and consent to participate**: Approved ethical clearance letter was obtained from Addis Ababa University, College of Health Sciences, Department of Emergency Medicine Ethical Review Committee. A support letter was written to the administration of the study hospitals for grant permission to conduct the study and permission was obtained from each ICU directorate. Participants were informed verbally and those who were not volunteers had been permitted not to participate in the study. Informed written consent was obtained from respondents who had participated in the study. The voluntary nature of the study and privacy of the participants during the data collection was assured by conducting in a comfortable private place and their personal information was protected from the public and secured by the researchers.

## Operational Definitions

**Level of knowledge**: Each answer for knowledge questions was given a “1” score for correct answers and “0” for incorrect answers. The total score was sixteen and it was then converted to a percentage and interpreted as follows. Those who scored <65% were considered as having inadequate knowledge whereas those who scored ≥ 65% were having adequate knowledge.

**Level of practice**: Practice has six Likert scale questions; for never, sometimes and almost always, a “0” score was given whereas for always “1” score was given. Then, all values were converted to percentages and interpreted as follows. A total score of <70% were considered as having a poor practice whereas a score of ≥70% were considered as having good practice.

## Result

**Socio-demographic characteristics of respondents**: The current study included 192 participants with a response rate of 98% whereas four questionnaires were not returned. Most of the respondents (51.6%) were female and their ages ranged from 20 to 50 years old with a mean age of 27.96 (SD ± 4.71). A majority of the respondents, 169(88.0%) had a Bachelor of Science degree in nursing, and 129(67.2%) had been employed at a critical care unit for more than a year (Table 1).

**Table 1:** Socio-demographic characteristics of the respondents, 2020 (N=192)

Variables(n=192)	Categories	Frequency	Percentage (%)
Sex	Female	99	51.6
	Male	93	48.4
Age	20–28	135	70.3
	29–34	39	20.3
	35–45	16	8.3
	≥46	2	1.0
Educational status	Diploma	12	6.3
	First degree	169	88.0
	Second degree	11	5.7
Work experience in ICU	<1Year	63	32.8
	≥1 Year	129	67.2

**Other characteristics of respondents**: Most of the participants 146 (76.0%) replied that enteral nutrition training was incorporated into their nursing school courses, while most of the participants 140 (72.9%) mentioned receiving no training in enteral nutrition during their career.

**Knowledge of the study participants on enteral nutrition**: Participants were asked sixteen questions about enteral nutrition in the ICU in order to assess their knowledge. They were categorized into two groups based on their responses to each question. According to the results, approximately two-thirds 130 (67.7%) of respondents had inadequate knowledge; however, 62 (32.3%) of respondents had adequate knowledge.

Approximately 104 (54.2%) of study participants were unaware that guidelines existed in their ICU, and the majority 116 (60.4%) said that their ICU department has no EN protocol. In most cases, 139 (72.4%) respondents knew which route to use to administer nutritional supplements. There were 184 participants in the study (95.8%) who answered incorrectly about the amount of gastric residual volume to be withheld, and most of the participants had an inadequate understanding of the absolute contraindications of EN (Table 2).

**Table 2:** Knowledge score of the participants, 2020 (N=192)

Variables	Correct	Incorrect
	
	Frequency(%)	Frequency(%)
Awareness of participants on enteral feeding guideline	88(45.8%)	104(54.2%)
Availability of enteral nutrition protocol in ICU	76(39.6%)	116(60.4%)
The preferable route of providing nutrition in ICU unless contraindicated.	139(72.4%)	53(27.6%)
The time for initiation of enteral nutrition unless contraindicated.	181(94.4%)	11(5.6%)
The absence of bowel sound is a complete contraindication for enteral nutrition.	44(23%)	148(77%)
Passage of flatus is a must prior to initiating enteral nutrition.	68(35.4%)	124 (64.6%)
Insertion and confirm Ryle's tube position in your ICU.	4(2.2%)	188(97.8%)
Supplying Ryle's tube feed in your ICU.	136(70.8%)	56(29.2%)
Amount of residual gastric volume for Ryle's tube feeds to be withheld.	8(4.2%)	184(95.8%)
The time for discarding supplied bottle feed (if left unused).	78(40.7%)	114(59.3%)
**Absolute contraindications to EN**		
Bowel obstruction	40 (20.8%)	152(79.2%)
Paralytic illus.	15 (7.8 %)	177(92.2%)
Intestinal ischemia/severe shock	8 (4.2%)	184(95.8%)
Severe GI bleeding	23(12%)	169(88%)
Generalized peritonitis	71(37.0%)	121(63.0%)
Intractable vomiting	7(3.6%)	185(96.4%)

**Practice of the study participants on enteral nutrition**: In assessing their level of practice about enteral feeding in the ICU, participants were asked six questions. According to the results of the current study, more than half 103 (53.8%) respondents had poor enteral feeding practices, while only 89 (46.4%) had good practices. One hundred fourteen (59.4 %), 115 (59.9 %), and 98 (51.0 %) of the participants responded that they always confirm tube placement before delivery of feeding, flushing of the tube before and after administration of feeding, and documenting any nutritional support or complication about their patient respectively whereas 117(60.9 %), 125 (65.1 %) and 129 (67.2 %) of them were not always participating in checking gastric residual volume before initiate feeding, conducting a daily inspection of nostrils and discussing nutritional management of patients during ward rounds respectively (Table 3).

**Table 3:** Participants' level of practice in Likert scale, 2020(N=192)

Variables	Always	Others
Confirming tube placement before delivery of feed.	114(59.4 %)	78(40.6 %)
Flushing of the tube before and after administration of feed.	115(59.9 %)	77(40.1 %)
Checking gastric residual volume before initiating feed.	75(39.1 %)	117(60.9 %)
Conducting a daily inspection of nostrils.	67(34.9 %)	125(65.1 %)
Document any nutritional support or complication about your patient.	98(51.0 %)	94(49 %)
Discussing nutritional management of patients during ward rounds.	63(32.8 %)	129(67.2 %)

**Figure 2:**
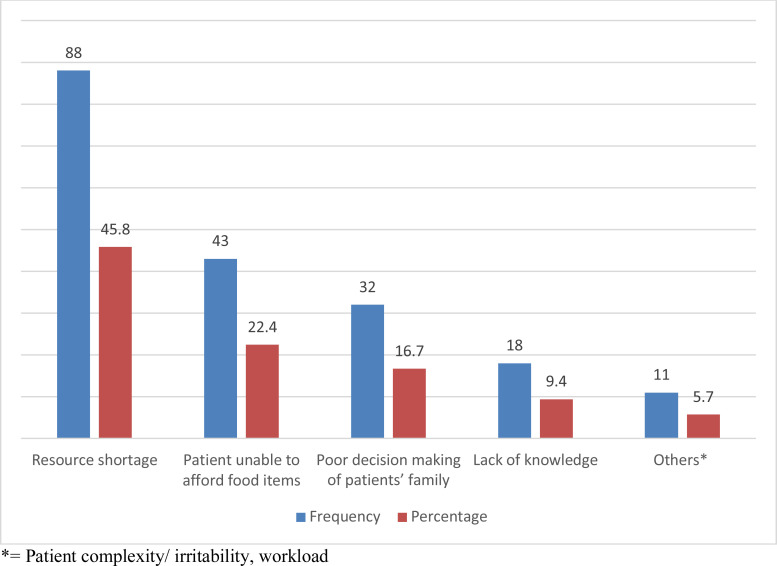
Challenges facing ICU nurses towards enteral nutrition, 2020 (N=192).

**Challenges of nurses towards effective nutritional support**: Among the challenges nurses face in providing nutritional support in ICU, lack of resources accounted for 45.8%, followed by inability of patients to afford food items, which accounted for 22.4% (Figure 2).

**Bivariate and multivariable analysis of factors affecting level of knowledge and practice of the participants**: As a result of bivariate analysis, females were 0.48 times less likely to have adequate knowledge (COR= 0.48, 95% CI: 0.19, 1.16) than males, and on multivariable analysis, nurses with BSC degrees were 0.24 times less likely to possess adequate knowledge (AOR = 0.24, 95% CI: (0.61, 0.93)) than nurses with MSC degrees. As for the practice of respondents, those in the age group of 20–28 were 0.02 times less likely to have a good practice on enteral nutrition (AOR = 0.02, 95% CI: (0.001,0.52)) than those in the age group of 46–50, and respondents who participated in school nutritional training were about 2 times more likely to have a good practice on enteral nutrition (AOR = 1.95, 95% CI: (0.06, 0.60)) than those didn't get the training. Also, participants who had awareness of enteral nutritional protocol were about 3 times more likely to have a good practice (AOR =3.40, 95% CI: (1.18, 9.78) than those who had no awareness (Table 4).

**Table 4:** Bivariate and multivariate analysis of factors affecting knowledge and practice of the participants on enteral nutrition, 2020 (N=192)

Variable	Categories	Level of Knowledge		COR (95% C.I)	AOR (95% C.I)
				
		Adequate	Inadequate		
Sex	Female	9	90	0.48(0.20, 1.15) *	0.47 (0.19, 1.15)
	Male	16	77	1.0	1.0
Educational status	Diploma	2	9	0.35(0.05, 2.46)	0.40(0.50,3.27)
	Degree	19	150	0.22(0.05, 0.82) *	0.24(0.61,0.93) **
	Masters	4	7	1.0	1.0
		**Level of practice**			
				
		**Good**	**Poor**		
Age	20–28	12	123	0.09 (0.006,1.66) *	0.02(0.001,0.52) **
	29–34	8	31	0.25 (.01,4.59)	0.12(0.006,0.2.83)
	35–45	0	16	<001	<001
	≥46	1	1	1.0	1.0
In school training	Yes	12	134	0.36 (0.14,0.94) *	1.95(0.06,0.60) **
	No	9	37	1.0	1.0
Patient unafforded the	Yes	6	81	0.44 (0.16,1.20) *	0.38 (0.10,1.33)
resources	No	15	90	1.0	1.0
In service training on	Yes	5	56	0.64 (0.22,1.84) *	0.81(0.23,2.81)
enteral nutrition	No	16	115	1.0	1.0
Resource supply	Yes	14	90	1.8 (0.69,4.68) *	1.90(0.64,5.65)
	No	7	81	1.0	1.0
Availability of protocol	Yes	13	63	2.78 (1.09,7.08) *	3.40(1.18,9.78) **
	No	8	108	1.0	1.0

COR*= Crude odds ratio significant at P<0.25 and AOR**=Adjusted odds ratio significant at P<0.05

## Discussion

According to the findings, almost two-thirds of the respondents 130 (67.7%) had insufficient understanding, while just 62 (32.3%) had acceptable knowledge of enteral nutrition. The findings were consistent with a study conducted in Pakistan, which indicated that just 10% of participants had appropriate levels of expertise (22). The findings were similarly incongruent with those of Al Kalaldeh (2015), who evaluated 253 critical care nurses from three major Jordanian hospitals; the results revealed that almost 70% of the participants scored less than 60% in enteral nutrition knowledge understanding (23). This was also similarly comparable to a study conducted in Alexandria, Egypt, where just 15 nurses (17.6%) out of 85 possessed adequate understanding (24).

One hundred and three participants (53.8%) had poor practice, whereas 89 (46.3%) had a good practice. Enteral nutrition in Addis Ababa ICUs is based on views rather than evidence-based approaches. This conclusion was consistent with research conducted at Egypt's Ismailia General Hospital, which found that more than half of the nurses examined had an unacceptable level of skill when it came to providing care prior to NG tube feeding administration (25).

In terms of participant awareness of guideline availability, 104 (54.8%) of study participants were unaware that a guideline was accessible in their ICU, and the majority of them 116 (60.4%) said their ICU lacked a procedure. This was countered by research conducted in Australia, which found that following procedures improved enteral feeding delivery and improved clinical outcomes in critically ill patients (26). The majority of the people polled said they didn't have access to an enteral nutrition protocol to help them supply nourishment. This conclusion corresponded to research conducted in Pakistan, which found that only a few nurses performed adequately in this area (22).

All facilities are supposed to provide safe nutritional assistance based on guidelines and protocols, but only 76 (39.6%) and 88 (45.8%) of the participants knew about EN protocols and guidelines, respectively. This contradicted Hyland et al findings which showed that procedure can greatly increase nutritional support (11).

According to the findings, the most significant factor contributing to unintentionally underfeeding habits in our study was a lack of resources, which hampered 45.8% of participant's ability to provide optimum enteral nutrition, followed by families' inability to provide nutritional support (22.4 %). Participants recognized barriers modestly, with a higher emphasis on insufficient resources in the ICU, which was consistent with a study conducted in Jordan (21).

The majority of study participants said that a lack of feeding tubes, a lack of understanding, and work overload were primary obstacles they faced during enteral nutrition procedures. The findings were similar to those of a study conducted in Egypt's Ismailia general hospital, which found that factors affecting nurses' practice regarding nasogastric tube feeding included a lack of learning, physical exhaustion, stress from being contaminated, a lack of nursing staff, reduced pay, non-appearance defensive garments, increased workload, and no incentives or redesigns for effective medical attendants (25).

Multivariable regression demonstrated no statistically significant difference in enteral nutrition knowledge and practice between males and females in this study. This result matched the findings of AlKalaldeh's (2015) study, which looked at nurses from three Jordanian hospitals and found no significant differences between male and female nurses in terms of knowledge and practice (24).

This finding demonstrated that nurses' educational status was closely linked to their understanding of enteral nutrition. Participants with a BSC degree were 0.24 times less likely than MSC degree holders to have appropriate knowledge (AOR = 0.24, 95 % CI: (0.61, 0.93)) in multivariable logistic regression. This conclusion contradicted a prior study conducted in Malawi, which found no statistically significant difference in knowledge between certificate nurses and state registered nurses. This disparity could be related to the fact that all Malawian nurses received inservice training on enteral feeding, which is not the case in our nation (18).

Study participants in the age group of 20–28 were 0.02 times less likely than the age group of ≥46 to have a good practice on enteral nutrition (AOR = 0.02, 95 % CI: (0.001,0.52). This study was not comparable to a study conducted in Egypt, which found that the majority of respondents were under the age of 30; however, this variable was not associated with any significant knowledge and skill results (22).

This study found that nurses who had enteral nutrition training in school were twice as likely as those who did not (AOR = 1.951, 95 % CI: (0.06, 0.60) to have good enteral nutrition practice. This was in line with research conducted in Egypt, which found that nurses who had previously attended knowledge-related educational sessions scored much higher than those who had not (25).

In conclusion, the nurses' knowledge and practices related to enteral nutrition in public hospitals in Addis Ababa, Ethiopia, were found to be inadequate, with certain dangerous procedures. Enteral nutrition was dependent on views rather than evidence-based methods in these ICUs.
